# Comparison of Gene Expression Profiles in Chromate Transformed BEAS-2B Cells

**DOI:** 10.1371/journal.pone.0017982

**Published:** 2011-03-18

**Authors:** Hong Sun, Harriet A. Clancy, Thomas Kluz, Jiri Zavadil, Max Costa

**Affiliations:** 1 Nelson Institute of Environmental Medicine, New York University School of Medicine, Tuxedo, New York, United States of America; 2 Department of Pathology, NYU Cancer Institute and Center for Health Informatics and Bioinformatics, NYU Langone Medical Center, New York, New York, United States of America; National Jewish Health, United States of America

## Abstract

**Background:**

Hexavalent chromium [Cr(VI)] is a potent human carcinogen. Occupational exposure has been associated with increased risk of respiratory cancer. Multiple mechanisms have been shown to contribute to Cr(VI) induced carcinogenesis, including DNA damage, genomic instability, and epigenetic modulation, however, the molecular mechanism and downstream genes mediating chromium's carcinogenicity remain to be elucidated.

**Methods/Results:**

We established chromate transformed cell lines by chronic exposure of normal human bronchial epithelial BEAS-2B cells to low doses of Cr(VI) followed by anchorage-independent growth. These transformed cell lines not only exhibited consistent morphological changes but also acquired altered and distinct gene expression patterns compared with normal BEAS-2B cells and control cell lines (untreated) that arose spontaneously in soft agar. Interestingly, the gene expression profiles of six Cr(VI) transformed cell lines were remarkably similar to each other yet differed significantly from that of either control cell lines or normal BEAS-2B cells. A total of 409 differentially expressed genes were identified in Cr(VI) transformed cells compared to control cells. Genes related to cell-to-cell junction were upregulated in all Cr(VI) transformed cells, while genes associated with the interaction between cells and their extracellular matrices were down-regulated. Additionally, expression of genes involved in cell proliferation and apoptosis were also changed.

**Conclusion:**

This study is the first to report gene expression profiling of Cr(VI) transformed cells. The gene expression changes across individual chromate exposed clones were remarkably similar to each other but differed significantly from the gene expression found in anchorage-independent clones that arose spontaneously. Our analysis identified many novel gene expression changes that may contribute to chromate induced cell transformation, and collectively this type of information will provide a better understanding of the mechanism underlying chromate carcinogenicity.

## Introduction

Hexavalent chromium [Cr(VI)] is widely used in numerous industrial processes, including chrome pigment production, chrome plating, stainless steel manufacturing, and leather tanning, etc. Epidemiological studies have reported a high incidence of lung cancer among chromium workers exposed occupationally to Cr(VI) by inhalation [1]–[3]. An early epidemiology study showed that 21.8% of deaths among chromium workers were due to respiratory cancer while only 1.4% of deaths could be attributed to respiratory cancer in the unexposed reference population [2]. The lung cancer risk among chromium workers was positively correlated with a longer duration of employment and with exposure to less water-soluble chromate compounds [2]. Numerous studies suggested that chromate induced DNA damage, mutation, genetic instability and epigenetic modulation of histones and DNA may contribute to its carcinogenicity, however, the molecular mechanisms of Cr(VI) induced lung cancer are not well understood.

Chromate can induce a wide variety of injuries in cells. After entering cells, Cr(VI) undergoes a series of metabolic reductions to form reactive Cr(V) and Cr(IV) intermediates as well as the final stable metabolite Cr(III) [4] [5]. These reactive intermediates and final products generated from the reduction process are able to induce the formation of stable Cr-DNA ternary adducts, protein-DNA cross-links, and DNA-DNA cross-links. These modifications, in combination with reactive oxygen species (ROS), may generate DNA single or double-strand breaks, which in turn may lead to mutations, chromosomal aberrations, and microsatellite instability [6] [7]. An increased frequency of microsatellite instability in Cr(VI)-induced lung tumors has been attributed to the ability of chromate to disrupt DNA mismatch repair [8] [9].

In addition to DNA damage, Cr(VI) is able to induce a broad range of changes in the epigenetic machinery. Chromium exposure of G12 Chinese hamster cells increased both genome-wide and gene-specific DNA methylation and silenced the expression of a gpt transgene [10]. In human lung cells, chromium exposure modulated histone methylation in both global and gene promoter-specific manner [11]. Interestingly, Histone H3 lysine 9 dimethylation, a silencing mark, was enriched in the human DNA mismatch repair *MLH1* gene promoter following chromate exposure and this was correlated with decreased *MLH1* mRNA expression [11]. Moreover, increased DNA methylation in the promoter region of *MLH1* gene and subsequent gene silencing were found in chromium-induced human lung tumors [8], suggesting epigenetic modulation as an important mechanism mediating Cr(VI)-induced lung carcinogenesis.

Cr(VI) induced tumorigenesis is thought to be a multistep process involving DNA damage, mutation, chromosome instabililty, aneuploidy, as well as epigenetic modulation [3]. The ultimate outcome of this process is the malignant cell phenotype that exhibited an altered gene expression profile. Previous studies have shown changes in gene expression following acute exposure of human cells to chromate (1 day or less), and identified a number of genes that were altered in response to acute chromate induced stress [12]–[14]. However, due to the complex effects of chromate in cells, changes noted in these early response genes may not play a role in cell transformation and tumorigenesis that arises with a latency period of at least a month. The purpose of our study was to identify genes that are characteristic of Cr(VI) induced cell transformation. Here, we employed a strategy to select cells for anchorage-independent growth following chronic chromate exposure. Our results showed that chronic exposure of immortalized normal human bronchial epithelial BEAS-2B cells to low doses of chromate promoted a high incidence of anchorage-independent growth. Interestingly, cell lines derived from soft agar colonies following chromate exposure exhibited altered morphology compared to cell lines derived from the untreated small colonies that arose spontaneously which morphologically resembled normal BEAS-2B cells. 409 differentially expressed genes were identified in Cr(VI) transformed cells compared to control cells, and these changes contributed to the functional and phenotypic differences between these two cell populations. It is of interest that gene expression profiles in six independent cell lines derived from Cr(VI) treated cells were very similar to each other, yet differed greatly from gene expression profiles in control cell lines derived from colonies that arose spontaneously in soft agar.

## Results

### Establishment of chromate transformed cell lines

**Figure 1:**
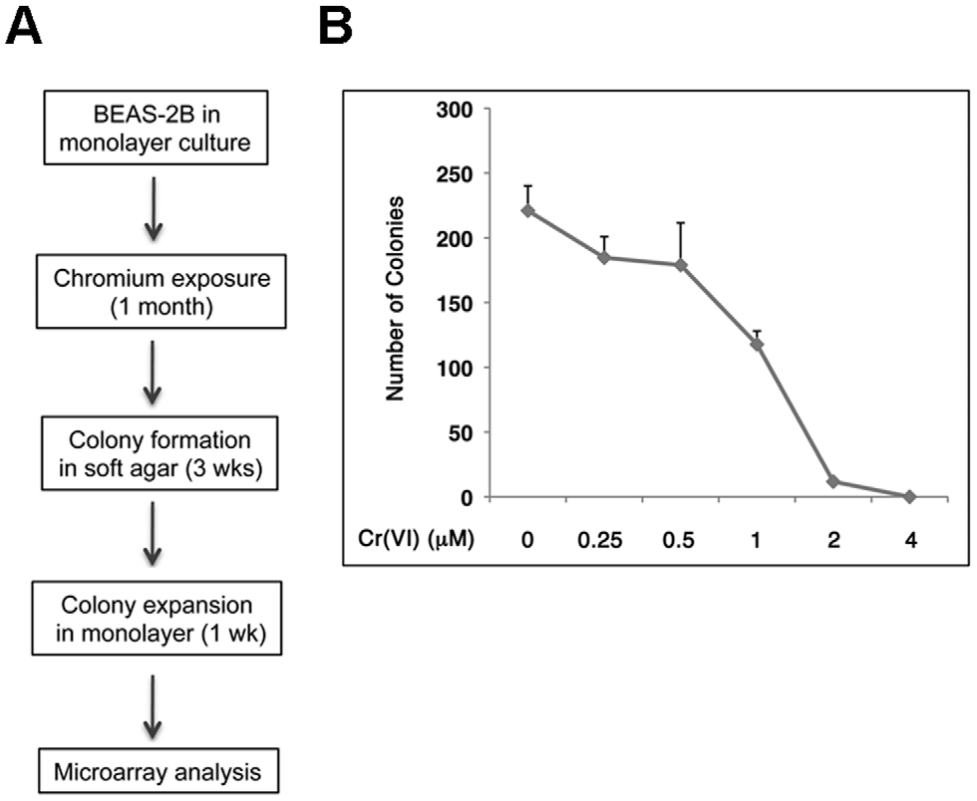
Effect of Cr(VI) on colony survival of immortalized human bronchial epithelial BEAS-2B cells. (**A**) Schematic presentation of the strategy to establish Cr(VI) transformed cell lines. (**B**) BEAS-2B cells were exposed to different doses of Cr(VI) (0.25, 0.5, 1.0, 2.0, and 4.0 µM) for 24 hours, and then subjected to colony survival assay in the absence of Cr(VI) for 2 weeks. Cell colonies were stained with Giemsa solution. The number of colonies was counted and presented as the mean ± SD (n = 3).

With the exception of cells from hematopoietic and lymphoid lineages, most normal cells rely on physical attachment to the extracellular matrix in order to manifest normal cell growth. In contrast, many transformed cells acquire the ability to grow and survive without attaching themselves to the substratum, a phenomenon commonly known as anchorage-independent growth. Due to its high correlation with tumor progression *in vivo*, anchorage independent growth has been considered a hallmark of cell malignancy and is used as a marker for *in vitro* cell transformation assays [15] [16]. In order to establish Cr(VI) transformed cell lines, we employed a strategy to select and sub-clone the transformed cells by taking advantage of their ability to grow in an anchorage-independent manner (**Figure 1A**).

**Figure 2:**
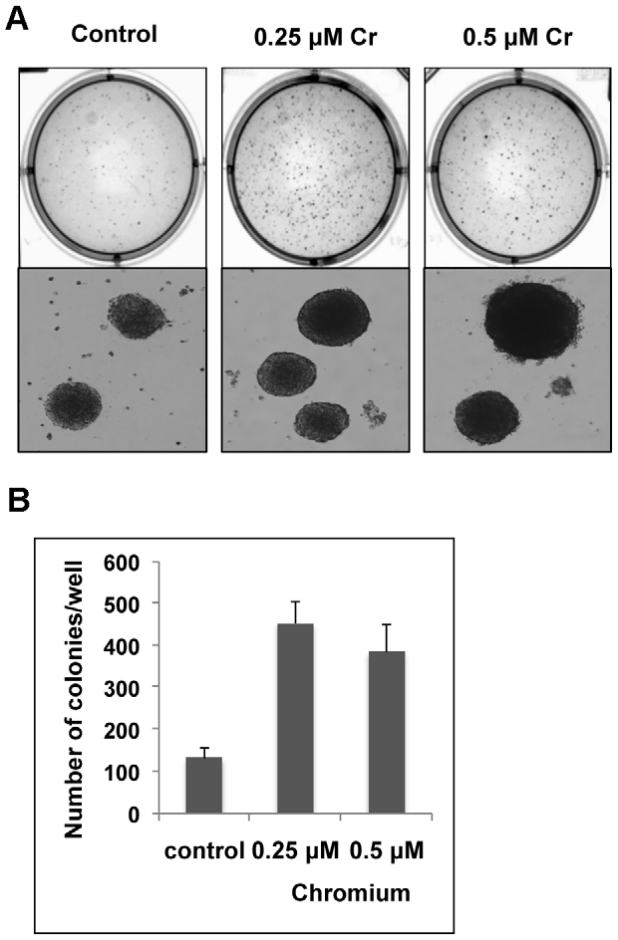
Chronic exposure of Cr(VI) promotes the anchorage-independent growth of BEAS-2B cells. BEAS-2B cells were exposed to 0.25 or 0.5 µM Cr(VI) for 1 month, and assessed for anchorage-independent growth using a soft agar assay. 3 weeks later, cell colonies were stained with INT/BCIP and photographed. (**A**) Representative plates in soft agar assay were shown. (**B**) Numbers of colonies per well were counted and presented as the mean ± SD (n = 4).

To mimic low dose and long term exposure *in vivo*, immortalized human bronchial epithelial BEAS-2B cells were continuously cultured in medium containing 0.25 or 0.5 µM of Cr(VI), doses in which BEAS-2B cells exhibited minimal toxicity (Figure 1B). During a 4-week exposure, cells were maintained in a sub-confluent state. While Cr(VI) treated cells exhibited a slower growth rate compared to untreated cells, no obvious difference in cell morphology was observed during the treatment (data not shown). After a 4-week exposure, anchorage-independent growth was assessed in both untreated and chromate treated BEAS-2B cells. Both Cr(VI) treated and untreated control BEAS-2B cells were grown in 0.35% top agar for three weeks. While there was a low level of background growth with untreated BEAS-2B cells, both 0.25 and 0.5 µM Cr(VI) treated cells formed significantly more colonies in soft agar (**Figure 2A**). Cells exposed to Cr(VI) demonstrated the greatest number of colonies that were three times more abundant compared to the untreated cells (**Figure 2B**). These results indicated that Cr(VI) exposure was able to significantly enhance anchorage-independent growth of BEAS-2B cells.

After 3 weeks of growth in soft agar, colonies reached a size that allowed them to be collected individually using a dissecting microscope. Five colonies were isolated from untreated colonies that arose spontaneously in soft agar and this group was designated as the control group. Ten colonies were selected from the 0.5 µM Cr(VI) exposure for further study. Five of these were large colonies and therefore designated as the Cr_large group, while the other five were selected to best match the size of the untreated control colonies and designated as Cr_small group. Isolated colonies were trypsinized and expanded in monolayer culture. Thus, each sub-cloned cell line contained a cell population derived from a single cell.

**Figure 3:**
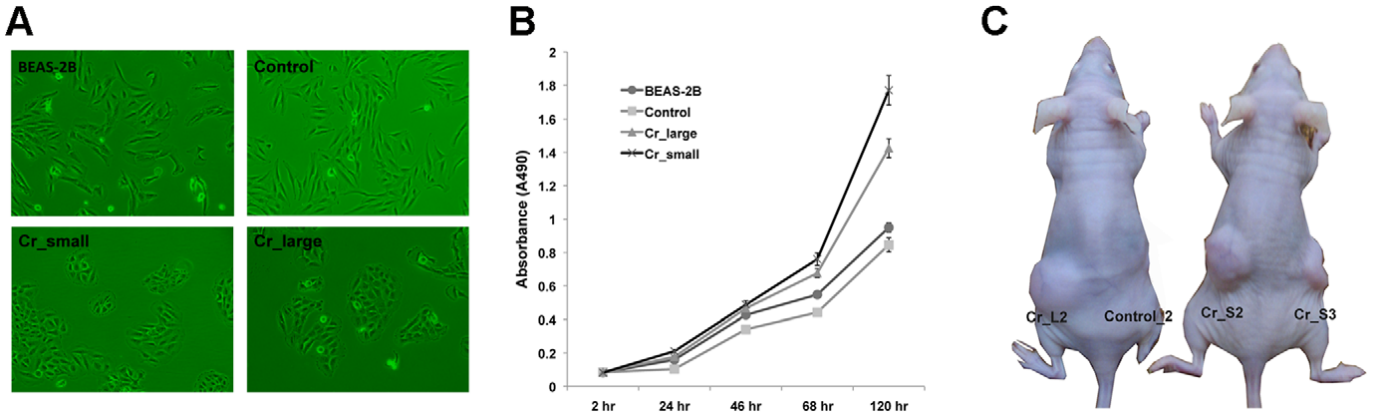
Distinct cell morphology of Cr(VI) transformed cells and tumor formation in nude mice. (**A**) Representative image of normal BEAS-2B cells (Beas-2B), control cells derived from spontaneously derived colonies of untreated cells (control), Cr(VI) transformed cells derived from large colonies (Cr_large) or small colonies (Cr_small) of 0.5 µM Cr(VI) treated cells grown in low density. (B) Normal BEAS-2B cells, control and Cr(VI) transformed cells were seeded at 2500 cells/well in 96-well plates. Cell numbers were measured by MTS assay at the indicated time point. Results were represented as mean ± SD (n = 6). (**C**) Representative image of tumor formation in nude mice after subcutaneously injection of control cells (control-2) and Cr-transformed cells derived from large colonies (Cr_L2) or small colonies (Cr_S2, S3).

After expansion in monolayer culture, Cr(VI) transformed cells exhibited distinct cell morphology compared to control cells. As shown in **Figure 3A**, control cells were flat, diamond-shaped, and similar to their parental BEAS-2B cells, while cells in both the Cr_large and the Cr_small groups were rounder, forming a more compact “cobblestone” monolayer. In addition, these Cr(VI) transformed cells easily detached from the culture surface when trypsinized, and exhibited a slightly faster growth rate as compared to control cells (**Figure 3B**). Moreover, 9 out of 12 mice injected with cells from Cr_large and Cr_small group developed tumors, while the mice injected with control cells did not form tumors after 6 months (**Figure 3C**). These data indicated that chronic exposure to Cr(VI) resulted in malignant transformation of BEAS-2B cells.

### Differential gene expression profiles in control and Cr(VI) transformed cells

Cr(VI) induced cell transformation is a multistep process in which cells go through an initial adaption to Cr(VI)-induced cell death and toxicity, then displayed anchorage-independent growth, and with time acquired the transformed phenotype. In order to characterize Cr(VI) transformed cells and identify the genes mediating Cr(VI) transformation, we analyzed the gene expression profiles in Cr(VI) transformed cell lines using microarray analysis. Three independent cell lines from each group (Cr_large, Cr_small, untreated control) were randomly chosen and subjected to microarray analysis using Affymetrix Human Gene 1.0 ST Array containing 28,869 well-annotated genes. Parental BEAS-2B cells were also included in this study.

**Figure 4:**
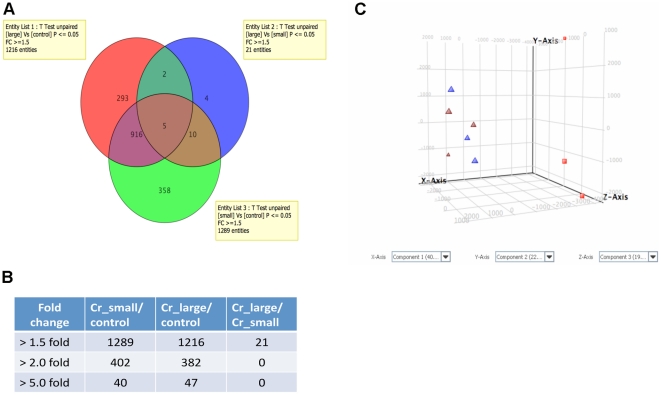
Gene expression profiles of control and Cr(VI) transformed cells. (**A**) Venn diagram showing the numbers of entities with more than 1.5-fold changed expression in pairwise comparison of Cr_large, Cr_small and control group. Numbers inside each compartment represent the number of entities. (**B**) The number of entities with more than 1.5-, 2-, and 5-fold changed expression level in pairwise comparison. (**C**) Principal Components Analysis revealed distinct separation between control cells and Cr(VI) transformed cells. Red square: control group; brown triangle: Cr_small group; blue triangle: Cr_large group.

First, we explored the microarray results by comparing gene expression profiles among Cr_large, Cr_small and untreated control groups. As shown in **Figure 4A**, a total of 1289 genes in the Cr_small group and 1216 genes in the Cr_large group displayed a greater than 1.5-fold difference as compared with the control group. When the cut-off threshold was increased to a 5.0-fold difference, the numbers decreased to 40 and 47 respectively (**Figure 4B**). Interestingly, when the Cr_small group and the Cr_large group were compared, only 21 genes whose expression changed more than 1.5-fold showed a statistically significant difference between the two groups (p<0.05, alpha level only), but none of these genes exhibited more than a 2.0-fold change, suggesting that cells from the Cr_large and the Cr_small group shared a very similar gene expression pattern. Principal Components Analysis (PCA) of the microarray data revealed a clear separation among samples from the control group and those from both Cr(VI) transformed groups (**Figure 4C**, square vs triangle), but not between the Cr_large and the Cr_small groups (**Figure 4B**, brown vs blue triangles). The size of the small colonies derived from the Cr(VI) treated cells were specifically selected to match the size of the control colonies, indicating that it is the Cr(VI) treatment and not the colony size that contributed to the observed differences in gene expression.

### Identification of differentially expressed genes in Cr(VI) transformed cells

Since the gene expression pattern from the Cr_large group and the Cr-small group were quite similar, a combined gene list from two groups was generated for further comparison with the untreated control. After elimination of the probe sets that represented unannotated genes, there were a total of 45 genes that changed more than 5-fold in 1 out of 2 groups in the Cr(VI) transformed cells compared with the control group, including 23 down-regulated and 22 up-regulated genes. The gene names and fold change of these two groups are listed in **Table 1**.

**Table 1:** Genes with more than 5-fold changed expression in Cr(VI) transformed cells.

Affymetrix ID	Gene Symbol	Gene Name	Fold change (Cr_large vs. control)	Fold change (Cr_small vs. control)
8022692	DSC3	desmocollin 3	36.99	40.20
8069676	ADAMTS1	ADAM metallopeptidase with thrombospondin type 1 motif, 1	16.80	28.63
8174513	CHRDL1	chordin-like 1	14.05	18.21
8037272	PSG5	pregnancy specific beta-1-glycoprotein 5	10.33	9.21
8037267	PSG2	pregnancy specific beta-1-glycoprotein 2	10.32	8.06
8072626	TIMP3	TIMP metallopeptidase inhibitor 3	9.43	7.55
8037251	PSG7/8/4	pregnancy specific beta-1-glycoprotein 7/8/4	9.41	8.41
7961142	OLR1	oxidized low density lipoprotein (lectin-like) receptor 1	9.29	4.89
8169949	RP6-213H19.1	serine/threonine protein kinase MST4	8.94	6.11
8022711	DSC2	desmocollin 2	8.00	7.03
7912520	NPPB	natriuretic peptide precursor B	6.80	4.71
8037231	PSG3	pregnancy specific beta-1-glycoprotein 3	6.73	6.15
8112274	ELOVL7	ELOVL family member 7	6.04	4.72
8059279	EPHA4	EPH receptor A4	5.86	5.52
8168589	ZNF711	zinc finger protein 711	5.67	3.97
8129880	PERP	PERP, TP53 apoptosis effector	5.61	3.11
8015268	KRT34	keratin 34	5.36	3.66
8152506	SAMD12	sterile alpha motif domain containing 12	4.79	5.96
8063536	TFAP2C	transcription factor AP-2 gamma	4.74	5.10
8058091	SATB2	SATB homeobox 2	4.68	5.59
8069689	ADAMTS5	ADAM metallopeptidase with thrombospondin type 1 motif, 5	4.52	5.45
8037240	PSG1	pregnancy specific beta-1-glycoprotein 1	3.65	5.37
7997642	CRISPLD2	cysteine-rich secretory protein LCCL domain containing 2	-5.02	-5.93
8176578	USP9Y	ubiquitin specific peptidase 9, Y-linked	−5.07	−5.50
8113073	ARRDC3	arrestin domain containing 3	−5.23	−4.66
8098204	CPE	carboxypeptidase E	−5.28	−4.34
8089145	ABI3BP	ABI gene family, member 3 (NESH) binding protein	−5.44	−4.74
8029779	IGFL1	IGF-like family member 1	−5.47	−6.36
8170648	BGN	biglycan	−5.58	−5.76
8067233	PMEPA1	prostate transmembrane protein, androgen induced 1	−5.98	−5.70
8157524	TLR4	toll-like receptor 4	−6.62	−8.58
8059580	DNER	delta/notch-like EGF repeat containing	−6.76	−6.34
8121277	AIM1	absent in melanoma 1	−6.91	−6.69
7902565	LPHN2	latrophilin 2	−7.64	−3.69
8051583	CYP1B1	cytochrome P450, family 1, subfamily B, polypeptide 1	−7.87	−3.65
8176384	ZFY	zinc finger protein, Y-linked	−7.95	−8.34
8177222	CD24	CD24 molecule	−11.36	−9.99
8097628	HHIP	hedgehog interacting protein	−11.53	−12.45
7981986	SNRPN	small nuclear ribonucleoprotein polypeptide N	−11.73	−11.80
8104746	NPR3	natriuretic peptide receptor C/guanylate cyclase C	−12.13	−13.03
8122150	EYA4	eyes absent homolog 4 (Drosophila)	−12.48	−12.72
8176719	EIF1AY	eukaryotic translation initiation factor 1A, Y-linked	−14.55	−15.00
8057620	COL5A2	collagen, type V, alpha 2	−19.76	−18.94
8176624	DDX3Y	DEAD (Asp-Glu-Ala-Asp) box polypeptide 3, Y-linked	−25.71	−26.58
8176375	RPS4Y1	ribosomal protein S4, Y-linked 1	−32.64	−32.95

Among genes up-regulated in chromate transformed cells, there were two major sub-groups. The first group was related to female reproduction, including 5 pregnancy specific beta-1-glycoproteins (PSG1, 2, 3, 5, and 7) that were clustered on human chromosome 19. The second group contained three genes (DSC2, DSC3 and Perp) required for the assembly of the desmosome complex, a cell-to-cell junction important for maintaining the structural integrity of the epithelia.

In contrast to the up-regulated genes, the down-regulated gene exhibited more diversification in functional categories. Most of down-regulated genes encoded proteins that were either localized in the plasma membrane or in the extracellular space. For example, a major sub-group of the down-regulated genes encoded proteins associated with a cell surface receptor that mediated cell signaling, including CD24, DDX3Y, BGN, CPE, DNER, HHIP, LPHN 2, and TLR4. Interestingly, among the most significantly down-regulated genes, RPS4Y1, DDX3Y, EIF1AY, ZFY and USP9Y, were located on the Y chromosome. The five PSG genes increased in Cr(VI) transformed cells were also localized in a cluster on chromosome 19. It is not clear whether these changes were gene-specific or loci-specific. Cr(VI) exposure has been shown to induce chromosome instability, including both numerical and structural aberrations, which might cause similar expression changes in multiple genes clustered in a chromosomal region. It is worth noting that all six Cr(VI) transformed cell lines exhibited similar changes in these two sub-groups of genes. Further analysis of chromosome damage and aberration as well as epigenetic mapping of these Cr(VI) transformed cells will help us to understand the underlying mechanism for these gene expression changes.

### Real time PCR Validation of gene expression

**Figure 5:**
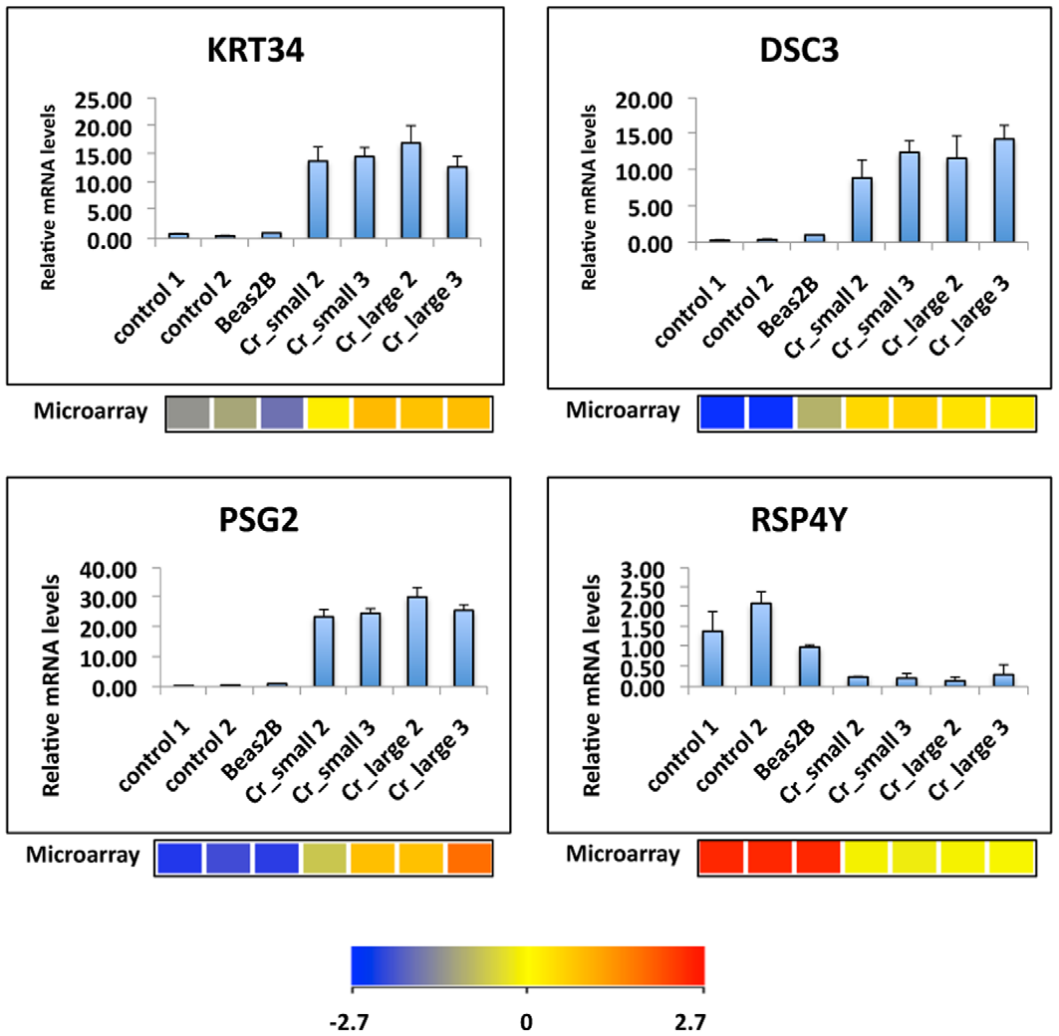
Validation of microarray results by quantitative RT-PCR. Total RNA was extracted from two cell lines each of control, Cr_small, and Cr_large group, as well as parental BEAS-2B cells. Expression levels of KRT34, DSC3, PSG2 and RSP4Y were analyzed by quantitative RT-PCR. Relative gene expression level, normalized to 18s rRNA expression, was presented as fold change to the level expressed in Beas-2B cells. Data are mean ± SD (n = 3). The expression value of each gene determined from the microarray data was listed below the corresponding PCR results. The color bar related color code to the expression value determined after quantile normalization and baseline transformation to the median levels of all samples.

To validate the results obtained from our microarray study, quantitative real-time PCR was performed on a subset of 4 genes exhibiting at least a 5-fold change in gene expression. Three up-regulated genes (KRT34, DSC3, PSG2) and 1 down-regulated gene (RSP4Y) were chosen based on their levels of expression in the microarray study. Differential expression of these genes in either Cr_large or Cr_small samples was validated using quantitative real-time PCR. As shown in **Figure 5**, the up- or down-regulated patterns for 4 genes obtained from real-time PCR were similar to those in the microarray study.

### Functional annotation and pathway analysis of differentially expressed genes

To assess the biological relevance of the differentially expressed genes, we performed the Gene Ontology (GO) annotation and pathway analysis using the DAVID functional annotation software [17]. The combined gene list from two Cr(VI) transformed groups exhibiting more than a 2-fold change in gene expression were used to identify the functional categories that were significantly over-represented (p<0.05) in Cr(VI) transformed cells. There were a total of 409 genes, including 142 up-regulated and 267 down-regulated genes (**Table S1**). GO term and KEGG pathways with significant over-representation were listed in **Table 2** (up-regulated genes) and **Table 3** (down-regulated genes). Among 142 up-regulated genes, the largest group with respect to both degree of significance (p<0.01) and number of genes was extracellular region (n = 22) in Cellular component, followed by cell motion (n = 10) in Biological process. The major pathways associated with up-regulated genes are p53 pathways (n = 3) and PPAR pathways (n = 3). Among 267 down-regulated genes, the largest group with respect to both degree of significance (p<0.001) and number of genes was extracellular region (n = 22) in Cellular component, followed by cell motion (n = 10) in Biological process. The major pathways associated with down-regulated genes were Focal adhesion (n = 12) and ECM-receptor interaction (n = 7). The following is a summary of genes changed in several major functional categories.

**Table 2:** Functional Annotation and pathway analysis of genes increased more than 2-fold in Cr(VI) transformed cells.

Category	GO Term	Number of genes	P Value
Biological Process	female pregnancy	8	0.0007
	cell migration	8	0.0020
	cell motion	12	0.0035
	reproduction	14	0.0102
	cell adhesion	11	0.0145
	cell differentiation	23	0.0158
	cell death	15	0.0172
	cell surface receptor linked signal transduction	20	0.0261
	blood circulation	5	0.0322
	fatty acid metabolic process	5	0.0391
	neurogenesis	9	0.0398
	folic acid transport	2	0.0443
	spermatid development	3	0.0474
Molecular Function	sugar binding	5	0.0279
	folic acid transporter activity	2	0.0290
	vitamin binding	4	0.0412
Cellular Component	desmosome	3	0.0066
	extracellular region	22	0.0099
	plasma membrane	33	0.0279
	cell junction	8	0.0408
	axon part	3	0.0437
KEGG Pathway	p53 signaling pathway	3	0.0910
	PPAR signaling pathway	3	0.0933

**Table 3:** Functional Annotation and pathway analysis of genes decreased more than 2-fold in Cr(VI) transformed cells.

Category	GO Term	Number of genes	P Value
Biological Process	wound healing	12	0.00002
	angiogenesis	8	0.00239
	cell motion	14	0.00600
	blood circulation	8	0.00828
	cell differentiation	32	0.00970
	regulation of cell growth	8	0.01029
	regulation of cell size	8	0.01397
	response to drug	8	0.01766
	cell adhesion	16	0.02637
	heterophilic cell adhesion	3	0.02969
	regulation of cell communication	21	0.03085
	transforming growth factor beta receptor signaling pathway	4	0.03776
	response to stress	30	0.03898
	respiratory system development	5	0.04454
	chromatin organization	10	0.04470
	epidermal growth factor receptor signaling pathway	3	0.04632
	elevation of cytosolic calcium ion concentration	5	0.04710
Molecular Function	carbohydrate binding	15	0.00014
	calcium ion binding	25	0.00044
	metallopeptidase activity	10	0.00047
	metal ion binding	69	0.00480
	actin binding	10	0.02074
	integrin binding	4	0.03687
	heparin binding	5	0.03940
	GTPase binding	5	0.04686
Cellular Component	extracellular matrix	13	0.00076
	plasma membrane	64	0.00108
	cytoplasm	106	0.00259
	extracellular space	18	0.00284
	chromatin	7	0.03015
	collagen type V	2	0.03467
	integrin complex	3	0.04491
	lamellipodium	4	0.04870
KEGG pathway	Focal adhesion	12	0.00041
	EMC-receptor interaction	7	0.00246

#### Changes in genes associated with cell junction

A major group of genes altered in Cr(VI) transformed cells were genes related to cell junction, a type of specialized structure mediating the contact between cells or between cells and extracellular matrix. In epithelial cells, there are four major types of cell-cell junctions: tight junction, gap junction, adherens junction and desmosomes. The major components of the desmosome complex, DSC2, DSC3, and Perp, are increased approximately 8-, 40- and 5- fold, respectively, in Cr(VI) transformed cells. In addition, CDH6, a type II classical cadherin involved in adherens junction, and CLDN1, the major protein for tight junction, are also up-regulated 4- and 2-fold, respectively, in Cr(VI) transformed cells. L1CAM, the L1 cell adhesion molecule, increased 4.7-fold in Cr(VI) transformed cells. Thus, genes associated with cell junction were up-regulated in Cr(VI) transformed cells.

#### Changes in genes associated with cell to extracellular matrix adhesion

In contrast to up-regulation of genes related to cell-to-cell contact, many genes involved in focal adhesion, a major type of cell junction mediating cell and extracellular matrix interaction, were decreased in Cr(VI) transformed cells. Integrins, the key component of focal adhesion, are transmembrane receptors that recognize and bind to most extracellular matrix proteins, such as collagens, fibronectin, and laminins [18]. Each integrin molecule is a heterodimer formed from 9 beta and 25 alpha subunits. Cr(VI) transformed cells exhibited decreased integrin alpha 5 (ITGA5), beta 3 (ITGB3) and beta-like 1 subunits (ITGBL1), but increased alpha 4 subunit (ITGA4). Similarly, the major component of the extracellular matrix, the ligands of the integrin receptor, were also decreased in Cr(VI) transformed cells: COL4A1, COL5A1, COL5A2, LAMB1 and LAMC2. Moreover, Fibulin-1 (FBLN1), an extracellular matrix protein often associated with fibronectin, which was able to inhibit the motility of a variety of cell types [19], decreased 3.6-fold in transformed cells. Fibrillin-1, a large extracellular matrix glycoprotein that sequestered TGFβ via an interaction with latent TGFβ binding protein [20], was also down-regulated in Cr(VI) transformed cells.

#### Additional changes in genes associated with extracellular matrix

ADAM metallopeptidase with thrombospondin type 1 molecules, ADAMTS-1 and ADAMTS-5, were up regulated 28- and 5.4-fold respectively in Cr(VI) transformed cells. It was reported that over-expression of ADAMTS-1 promoted pulmonary metastasis of TA3 mammary carcinoma and Lewis lung carcinoma cells [21], and forced expression of ADAMTS-5 in glioma cell lines stimulated cell invasion [22]. In addition, matrix metalloproteinase-2 (MMP-2), an enzyme which degraded type IV collagen, was decreased in Cr(VI) transformed cells. Simultaneously, its inhibitor, TIMP-3, was up-regulated more than 9-fold in the transformed cells.

#### Changes in genes associated with cell proliferation and growth

Despite the slightly faster growth rates observed in transformed cells, the expression of cell cycle related genes were quite similar in control and Cr(VI) transformed cells, with the exception of cyclin D1, which was increased about 2-fold in Cr(VI) transformed cells. Interestingly, dysregulation of cyclin D1 was found in 11 out of 16 (69%) chromium-induced lung SCC but only 3 out of 26 (12%) non-exposed lung SCC [23], indicating a specific connection between cyclin D1 overexpression and the lung cancer induced by chromate exposure. Thus, our finding of increased cyclin D1 in Cr(VI) transformed cells was consistent with the finding *in vivo*, supporting a crucial role of cyclin D1 in the development of chromate induced lung cancer.

In addition to genes directly involved in cell cycle progression, genes that regulated cell proliferation were also altered in expression. TGFβ signaling [24] and hedgehog signaling [25] are important pathways involved in regulating cell growth. We observed that there was decreased expression of TGFβ2 and TGFβR2 (2.4- and 3.5-fold respectively) in Cr(VI) transformed cells, suggesting that the loss of a cell response to TGFβ induced growth inhibition might be an early step of cellular transformation and tumorigenesis. Moreover, HHIP, a gene that antagonizes hedgehog signaling pathways, was decreased about 12-fold in Cr(VI) transformed cells.

#### Changes in genes associated with cell apoptosis

There are two major pathways controlling cell apoptosis. The extrinsic pathway involves the interaction of a death receptor including Fas and TNF receptor superfamily members and ligands, and the intrinsic pathway involves the mitochondria that operate in both p53-dependent and independent manner [26] [27]. Although the molecules involved in each pathway were quite different, both pathways lead to caspase activation and apoptosis. Several direct targets of p53 were increased in Cr(VI) transformed cells, including CYFIP2, Perp, and RNF144B, which were known to mediate p53-dependent apoptosis. MRPS30, a mitochondrial ribosomal protein associated with programmed cell death, was also up-regulated in transformed cells. In contrast to up-regulated genes related to intrinsic apoptosis, genes associated with extrinsic apoptosis pathways were slightly down-regulated in transformed cells. For example, NUAK2, a gene induced by FasL or TNF-alpha [28], was down-regulted 2-fold. It was previously reported that NUAK2 protects cells from FasL mediated cell apoptosis [29]. SEMA3A and RHOB, are also associated with the TNF and Fas pathways, and they decreased 4- and 3.1-fold, respectively. Within the caspase family member, only caspase 4 was found to be increased in Cr(VI) transformed cells.

### Identification of genes commonly expressed in both Cr(VI) treated and control cells

**Figure 6:**
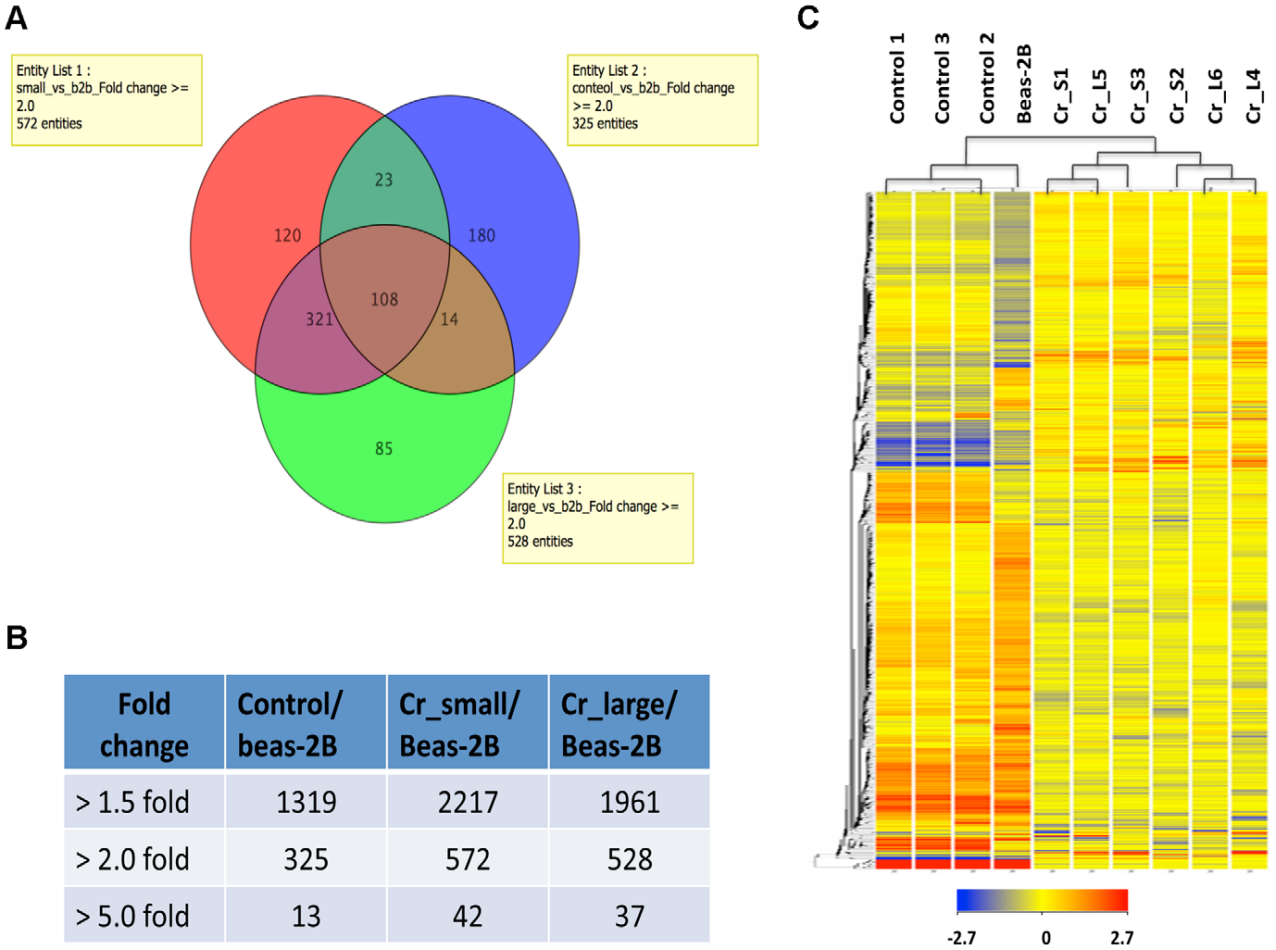
Gene expression profiles of control, Cr(VI) transformed and parental BEAS-2B cells. (**A**) Venn diagram showing the numbers of entities with more than 2-fold changed expression in pairwise comparison of Cr_large, Cr_small, control group and parental BEAS-2B cells. Numbers inside each compartment represent the number of entities. (**B**) The number of genes with more than 1.5-, 2-, and 5-fold changed expression level in pairwise comparison. (**C**) Hierarchical cluster analysis of genes with more than 2-fold changed expression in one out of three groups (control, Cr_small, Cr_large) compared to parental BEAS-2B cells. The color bar related color code to the expression value determined after quantile normalization and baseline transformation to the median levels of all samples.

Both Cr(VI)-transformed and control cells were derived from colonies that grew in soft agar. These cells underwent a change in growth that may have caused an alteration in gene expression. We next compared the gene expression profiles from cells derived from the soft agar colonies to their parental BEAS-2B cells. As shown in **Figure 6A**, there was a total of 851 genes changed more than 2-fold in 1 out of 3 groups compared to normal BEAS-2B cells, including 325 genes (38%) from the control group, 572 genes (67%) from the Cr_small group, and 528 genes (62%) from the Cr_large group. Similarly, within the 92 genes that changed more than 5-fold, only 13 genes (13%) are from the control group, compared to 42 (46%) and 37 (40%) genes in the Cr_small and the Cr_large groups, respectively (**Figure 6B**). Therefore, the differences between the Cr(VI) transformed cells and the control cells that grew in soft agar were more striking as compared to any differences caused by growth in soft agar.

Similar results can be seen by a hierarchical clustering analysis of 851 genes (**Figure 6C**), in which the samples were sorted based on the similarity of gene expression. The gene expression profiles of Cr(VI) treated cells were clearly separated from those in the control group as well as in parental BEAS-2B cells, however, no obvious separation can be seen among the six Cr(VI) transformed cell lines that were derived from colonies with different sizes. In contrast, control cells shared similar expression profiles with parental BEAS-2B cells and were clustered in the same group. It is of interest that the heat maps of gene expression were remarkably similar in six independently derived cell lines following Cr(VI) exposure. Additionally heat maps of control cell lines derived from spontaneously arose clones were also remarkably similar to each other, yet very different from those derived from Cr(VI) exposed cells.

It is worth noting that there were 108 entities shared by three groups as shown by the Venn diagram (**Fig. 6A**). 91 out of 108 entities represented well-annotated genes with similar changes in gene expression between control and Cr(VI) treated cells, which are likely the outcome of anchorage-independent growth. Functional annotation of these genes revealed an up-regulation of genes involved in the p53 signaling pathway and angiogenesis, as well as a down-regulation of genes associated with cell adhesion and cell growth inhibition. The complete list of 91 genes including 41 up-regulated and 50 down-regulated genes as well as the functional annotation can be found in Tables **S2** **and S3**.

### Loss of TGF-β1 responsiveness in Cr(VI) transformed cells

**Figure 7:**
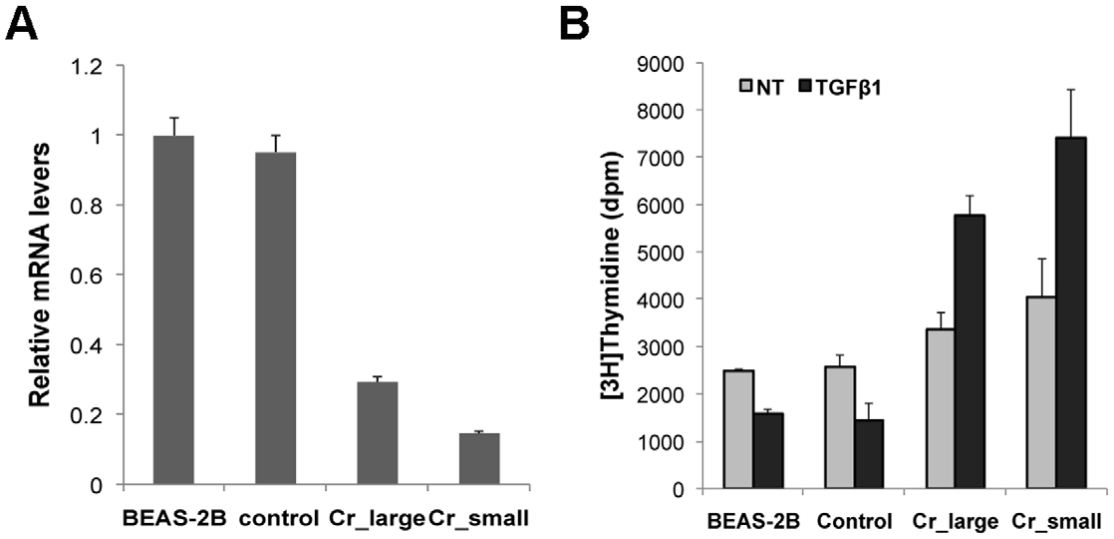
Reduced level of TGF-β type II receptor and resistance to TGF-β1 induced growth inhibition in Cr(VI) transformed cells. (A) Total RNA was extracted BEAS-2B, control, and Cr(VI) transformed cells. Expression levels of type II TGF-β receptor were analyzed by quantitative RT-PCR. Relative gene expression level, normalized to 18s rRNA expression, was presented as fold change to the level expressed in Beas-2B cells. (B) Normal BEAS-2B cells (Beas-2B), control cells (control), and Cr(VI) transformed cells (Cr_large, Cr_small) were seeded at 5000 cells/well in 96-well plates. Cells were then either left untreated or treated with 10 ng/ml TGF-β for 24 hr. Cell proliferation was measured by [^3^H]thymidine incorporation. Results were represented as mean ± SD (n = 3).

TGFβ family members regulate a wide range of biological processes including cell proliferation, migration, differentiation, apoptosis, and extracellular matrix deposition [24]. Previous reports showed that TGF-β1 can cause growth inhibition in normal human bronchial epithelial (NHBE) [30] and BEAS-2B cells [31], but not in human lung carcinoma A549 cells [30] as well as BEAS-2B cells overexpressing mutant p53 [31]. Resistance to TGFβ mediated growth inhibition in human lung cancer may occur through the loss of type II receptor (TGFβR2) expression. For example, it was reported that 77% NSCLC exhibited a reduced level of TGFβR2 [32]. Forced expression of TGFβR2 in lung cancer cells showed reduced colony formation in a soft agar assay as well as reduced tumorigenicity when injected into athymic nude mice [32]. Since the levels of TGFβR2 were down-regulated in Cr(VI) transformed cells (**Fig. 7A**), we tested whether these cells retained their ability to response to TGF-β1 induced growth inhibition. Control and Cr(VI) transformed cells as well as BEAS-2B cells were exposed to 10 ng/ml of recombinant human TGF-β1 for 24 hours. DNA synthesis were measured by [^3^H]thymidine incorporation. Consistent with previous reports, thymidine incorporation in BEAS-2B and control cells was reduced by 30% and 40% respectively in the presence of TGF-β1. However, Cr(VI) transformed cells from both Cr_large and Cr_small colony groups exhibited an increased DNA synthesis after exposing to TGF-β1 (**Fig. 7B**), suggesting that these transformed cells were able to escape from TGFβ1 induced growth arrest. These results further support that the loss of a cell response to TGFβ induced growth inhibition is acquired during cellular transformation and tumorigenesis.

## Discussion

Cr(VI) is a well established carcinogen that induced respiratory cancer and several other types of human cancer [2]–[3]. However, the molecular mechanism underlying Cr(VI) induced lung cancer and the down-stream genes that mediated Cr(VI) carcinogenicity are not complete understood. In the present study, we showed that chronic exposure of immortalized normal human bronchial epithelial BEAS-2B cells to low doses of Cr(VI) significantly enhanced their ability to grow in an anchorage-independent manner as assessed by increased number and size of soft agar colonies. In addition, the cell lines derived from the Cr(VI) exposed colonies exhibited striking changes in cell morphology and were able to form tumors when injected into nude mice, indicating that Cr(VI) was capable of inducing malignant transformation in BEAS-2B cells. Moreover, gene expression analysis revealed a change in expression profile in Cr(VI) transformed cells that may facilitate cell growth and migration. Interestingly, the gene expression profile of six Cr(VI) transformed cell lines were remarkably similar to each other, yet very different from those of the control cell lines, indicating that altered gene expression was indeed the consequence of chronic Cr(VI) exposure.

Two recent studies have reported the effect of chronic Cr(VI) exposure on BEAS-2B cells. Rodrigues et al. reported the establishment of Cr(VI) transformed cells by ring-cloning BEAS-2B cell after chronic exposure of cells to 1 µM Cr(VI) [33]. These transformed cells exhibited an aneuploid phenotype, up-regulation of genes associated with malignant transformation and DNA repair, as well as the ability to form tumors in nude mice [33]. Our present study confirmed their finding that chronic exposure to Cr(VI) could result in malignant transformation of BEAS-2B cells. However, we were not able to detect any changes in genes associated with DNA repair as reported by Rodrigues et al. The difference is likely due to the strategies used to establish transformed cell lines. In Rodrigues' study, cells were continuously exposed to Cr(VI) after the transformed clones were derived [33]. In our study, Cr(VI) exposure was terminated after 30 days. Cells were then allowed to form colonies in soft agar and subsequently expanded in monolayer in the absence of Cr(VI). Therefore, cells in their study were constantly exposed to Cr(VI) that activated a response to DNA damage and expressed genes related to DNA repair. Clearly, this was not the case in our study. A lack of continuous exposure to Cr(VI) may also explain why there were very few changes in genes related to oxidative stress in our transformed cells.

Costa et al reported that chronic exposure of BEAS-2B cells to sub-cytotoxic Cr(VI) resulted in dramatic changes in cell morphology and growth patterns [34], that was in agreement with our observation. Cr(VI) treated and transformed cells were more sensitive to trypsinization, suggesting extracellular matrix adhesion is a major target of Cr(VI). The phenotypic change of transformed cells was further supported by alteration in expression of genes during Cr(VI) induced cell transformation. The genes associated with cell to matrix adhesion were significantly down-regulated in Cr(VI) transformed cells, including many genes involved in focal adhesion. Decreased gene expression of integrin receptors, ligands, and other matrix components could result in a poor connection between cells and the substratum, that in turn could have contributed to a transformation phenotype and further facilitated cell migration and invasion.

It was interesting that genes related to cell junction were increased in Cr(VI) transformed cells. Genes belonging to this category were normally down-regulated during tumorigenesis to free tumor cells from the surrounding cell contacts and facilitate tumor invasion and metastasis. However, dysregulation of cell junction genes were found in a number of human cancers, and were required for tumor cell motility and invasion. For example, up-regulation of CHD6 has been observed in several types of human cancer, suggesting a possible role in metastasis and invasion [35]. L1CAM was reported to be involved in the advanced stages of tumor progression, and over expression of L1CAM in normal and tumor cells increased cell motility and metastasis [36]. Interestingly, both CHD6 and L1CAM were increased more than 4-fold in Cr(VI) transformed cells, suggesting a possible role of cell junction related genes in Cr(VI) induced cell transformation.

It is worth noting that our study demonstrated a dramatic increase in genes associated with desmosome complex in Cr(VI) transformed cells. Desmosome is a intracellular junction mediating the interaction between adjacent epithelials. Desmocollins (DSC1, DSC2 and DSC3) are members of the cadherin family, which form the transmembrane core of the desmosome through heterotypic interaction with another member of the same family, desmogleins (DSG) [37] [38]. Depletion of DSC3 in mice resulted in embryonic lethality before implantation [39], while conditional depletion of DSC3 in mouse epidermis led to epidermal blistering [40]. Perp was originally known as an apoptosis-associated target of p53. Recent work on Perp null mice indicate that Perp is crucial for desmosome formation as the *Perp* deficient mice died post-natally with dramatic blistering similar to DSC1 null mice [41]. Recently, DSC2 and DSC3 were found increased in human lung cancer, especially in lung squamous cell carcinoma [42]. DSC3 was considered as a marker for squamous carcinoma due to its high levels in almost all lung squamous carcinoma [43]. Interestingly, the majority of lung cancers in chromium workers were squamous cell carcinoma. Our finding that DSC3 increased more than 40-fold in Cr(VI) transformed cells was consistent with its high level in tumor cells. Two other components of desmosome, DCS2 and Perp, were also up-regulated in Cr(VI) transformed cells. It is possible that these desmosome genes were direct targets of chronic Cr(VI) exposure, and their up-regulation by Cr(VI) may contribute to its carcinogenicity. In addition, genes related to cell cycle control as well as cell proliferation were also changed. The level of cyclin D1, an important cyclin expressed in early G1 phase and required for cell cycle progression, was increased in Cr(VI) transformed cells. Dysregulation of cyclin D1 was frequently found in the early stage of tumorigenesis in many different cancers, and has been reported at high levels in chromate induced lung cancers. TGFβ is an important negative regulator of lung epithelial cells, and loss of TGFβ signaling is an early event that contributes to cell growth. Cr(VI) transformed cells exhibited significantly reduced levels of TGFβ2 and TGFβR2, and were able to escape from TGFβ induced growth inhibition. These results suggested that Cr(VI) may have promoted tumor cell growth by stimulating proliferation associated genes and inhibiting anti-proliferation genes.

In summary, our studies analyzed gene expression profiles in transformed cells lines expanded from a single colony in soft agar after chronic exposure to low doses of Cr(VI). We have identified many novel changes in gene expression that were different from the immediate response genes identified from previous studies using acute exposure of Cr(VI). These genes may be involved in Cr(VI) induced malignant transformation. Thus, further analysis of these genes *in vivo* and dissecting the mechanism of Cr(VI) induced expression alterations will provide a better understanding of the mechanism underlying chromium carcinogenicity.

## Materials and Methods

### Cell Culture

Human normal bronchial epithelial BEAS-2B cells [44] were cultured in DMEM (Invitrogen) supplemented with 10% FBS and 100 U/ml penicillin and 100 µg/ml streptomycin (Invitrogen). The cells were cultured at 37°C in an incubator with a humidified atmosphere containing 5% CO_2_. For Cr(VI) exposure, cells were treated with 0.25 or 0.5 µM potassium chromate (K_2_CrO_4,_ J. T. Baker Chemical Co.) for 1 month. The medium was changed every other day, and cells were split in the presence of Cr(VI) every 3 days.

### Colony survival assay

BEAS-2B cells were treated with various doses of Cr(VI) (0.25, 0.5, 1.0, 2.0, and 4.0 µM) for 24 hours. Control and Cr(VI) treated cells were plated at 500 cells/dish in 100-mm cell culture dishes, and cultured for two weeks. Cells were stained with Giemsa solution, and the number of colonies was counted and presented as mean ± SD (n = 3).

### Cell proliferation assay

Normal BEAS-2B cells, control cells derived from spontaneously formed colonies of untreated cells, and Cr(VI) transformed cells derived from large colonies (Cr_large) or small colonies (Cr_small) were seeded at 2500 cells/well in 96-well plates. Viable cell number was determined using the MTS CellTiter 96 aqueous one solution cell proliferation assay (Promega) according to the manufacturer's instructions.

### [^3^H]Thymidine incorporation assay

Cells were seeded at density of 5×10^4^ cells/well in 24-well plate. On the following day, cells were treated with 10 ng/ml recombinant human TGF-β1 (R&D System) for 24 hr. Cells were then pulse labeled with 0.5 µCi of [^3^H]thymidine for 2 hr. The cells were washed twice with ice cold PBS, followed by precipitated with ice-cold 5% TCA twice, each with 10 minutes. The cells were then extracted with 0.5 ml of 0.5 M NaOH for 2 hours at room temperature, and neutralized with 0.25ml of 1N HCl. The cell lysate was transferred to scintillation vials with 5 ml of scintillation fluid, and the radioactivity was assessed using a Scintillation Counter.

### *In vivo* tumorigenesis

Six-week old female athymic nude mice (NCI, Frederick) were injected subcutaneously at the left or the right flank with control cells and Cr transformed cells (5×10^6^ cells/0.1 ml/injection). Each cell line was injected into 3 mice. Six months later, the mice were photographed and sacrificed. Tumors and other tissues were collected for further analysis.

### Soft-agar assays

Control and Cr(VI) treated cells were plated at 5000 cells/well in 6-well plates with culture medium containing 0.35% low-melting-point agarose over a 0.5% agarose base layer and cultured at 37°C incubator with 5% CO_2_ for 3 weeks. The colonies were stained with INT/BCIP (Roche) and photographed.

### Microarray hybridization and data analysis

Total RNA from each cell line was extracted using Trizol (Invitrogen) according to the manufacturer's protocol. cRNA probes were synthesized and labeled using GeneChip Whole Transcript cDNA Synthesis and Amplification Kit and Terminal Labeling Kit (Affymetrix), and were subjected to hybridization with GeneChip Human Gene 1.0 ST Array (Affymetrix) that contains 28,869 well annotated genes. Hybridization and scanning of the arrays was performed using a standard procedure. Microarray data analysis was performed using GeneSpring v11 (Agilent Technologies). All microarray data is MIAME compliant and the raw data has been deposited in NCBIs Gene Expression Omnibus (GEO), and assigned Series accession number GSE24025. The expression value of each probe set was determined after quantile normalization using RMA16 algorithm and baseline transformation to the median levels of control samples. Differentially expressed genes were identified using an unpaired T test (p<0.05). Principal component analysis (PCA) was used to visualize the gene expression pattern of all samples. Hierarchical cluster analysis using Euclidean distance was performed to cluster genes and samples for heatmap. Functional annotation was analyzed with the Gene Ontology (GO) classification system using DAVID software (http://david.abcc.ncifcrf.gov/home.jsp).

### Real-time quantitative PCR

Total RNA was extracted from each cell line using TRIzol Reagent (Invitrogen), and converted to single stranded cDNA using Superscrip III (Invitrogen). Quantitative real-time PCR analysis was performed using SYBR green PCR system (Applied Biosystems) on ABI prism 7900HT system (Applied Biosystems). All PCR reactions were performed in triplicate. Relative gene expression level, normalized to 18s rRNA expression, was calculated by 2^−ΔΔCt^. The results were presented as fold change to the level expressed in BEAS-2B cells.

## Supporting Information

Table S1 **Complete list of 409 genes differentially expressed more than 2-folds in Cr(VI) transformed cells.**
(XLS)

Table S2 **Complete list of 91 genes shared similar expression changes in both Cr(VI) transformed and control cells.**
(XLS)

Table S3 **Functional annotation of 91 common genes between control and chromium transformed cells.**
(DOC)
