# Supplementary material for: Comparison of Gene Expression Profiles in Chromate Transformed BEAS-2B Cells
Source: PLoS One. 2011 Mar 18;6(3):e17982. doi: 10.1371/journal.pone.0017982 (PMC3060877; doi:10.1371/journal.pone.0017982)
Supplement: Table S3 — Functional annotation of 91 common genes between control and chromium transformed cells. (DOC) [file pone.0017982.s003.doc]

Table S3. Functional Annotation of 91 common genes between control and chromium transformed cells

| Category | GO Term | Number of genes | P Value |
| --- | --- | --- | --- |
| **Down-regulated genes** | | | |
| Biological Process | Negative regulation of cell proliferation | 6 | 0.0019 |
|  | cell adhesion | 6 | 0.029 |
| Molecular Function | carbohydrate binding | 4 | 0.044 |
|  | receptor binding | 7 | 0.025 |
| Cellular Component | Golgi apparatus | 7 | 0.042 |
|  | membrane | 28 | 0.018 |
|  | integral to membrane | 22 | 0.025 |
| KEGG Pathway | none |  |  |
| **Up-regulated genes** | | | |
| Biological Process | Vasculature development | 6 | 0.00033 |
|  | cell proliferation | 8 | 0.0025 |
|  | angiogenesis | 4 | 0.0055 |
|  | locomotion | 4 | 0.011 |
| Molecular Function | receptor binding | 6 | 0.021 |
|  | protein binding | 23 | 0.04 |
| Cellular Component | extracellular | 8 | 0.0037 |
|  | plasma membrane | 15 | 0.018 |
| KEGG_PATHWAY | p53 signaling pathway | 4 | 0.0019 |
|  | Axon guidance | 4 | 0.011 |
